# *NOTCH3* Variants and Risk of Ischemic Stroke

**DOI:** 10.1371/journal.pone.0075035

**Published:** 2013-09-23

**Authors:** Owen A. Ross, Alexandra I. Soto-Ortolaza, Michael G. Heckman, Christophe Verbeeck, Daniel J. Serie, Sruti Rayaprolu, Stephen S. Rich, Michael A. Nalls, Andrew Singleton, Rita Guerreiro, Emma Kinsella, Zbigniew K. Wszolek, Thomas G. Brott, Robert D. Brown, Bradford B. Worrall, James F. Meschia

**Affiliations:** 1 Department of Neuroscience, Mayo Clinic, Jacksonville, Florida, United States of America; 2 Section of Biostatistics, Mayo Clinic, Jacksonville, Florida, United States of America; 3 Center for Public Health Genomics, University of Virginia, Charlottesville, Virginia, United States of America; 4 Laboratory of Neurogenetics, National Institute on Aging, National Institutes of Health, Bethesda, Maryland, United States of America; 5 Department of Molecular Neuroscience and Reta Lilla Weston Laboratories, Institute of Neurology, London, United Kingdom; 6 Center for Neuroscience and Cell Biology, University of Coimbra, Coimbra, Portugal; 7 Cardiff University School of Medicine, Cardiff, United Kingdom; 8 Department of Neurology, Mayo Clinic College of Medicine, Jacksonville, Florida, United States of America; 9 Department of Neurology, Mayo Clinic, Rochester, Minnesota, United States of America; 10 Department of Neurology, University of Virginia, Charlottesville, Virginia, United States of America; Centre Hospitalier Universitaire Vaudois (CHUV), Switzerland

## Abstract

**Background:**

Mutations within the *NOTCH3* gene cause cerebral autosomal dominant arteriopathy with subcortical infarcts and leukoencephalopathy (CADASIL). CADASIL mutations appear to be restricted to the first twenty-four exons, resulting in the gain or loss of a cysteine amino acid. The role of other exonic *NOTCH3* variation not involving cysteine residues and mutations in exons 25-33 in ischemic stroke remains unresolved.

**Methods:**

All 33 exons of *NOTCH3* were sequenced in 269 Caucasian probands from the Siblings With Ischemic Stroke Study (SWISS), a 70-center North American affected sibling pair study and 95 healthy Caucasian control subjects. Variants identified by sequencing in the SWISS probands were then tested for association with ischemic stroke using US Caucasian controls collected at the Mayo Clinic (n=654), and further assessed in a Caucasian (n=802) and African American (n=298) patient-control series collected through the Ischemic Stroke Genetics Study (ISGS).

**Results:**

Sequencing of the 269 SWISS probands identified one (0.4%) with small vessel type stroke carrying a known CADASIL mutation (p.R558C; Exon 11). Of the 19 common *NOTCH3* variants identified, the only variant significantly associated with ischemic stroke after multiple testing adjustment was p.R1560P (rs78501403; Exon 25) in the combined SWISS and ISGS Caucasian series (Odds Ratio [OR] 0.50, P=0.0022) where presence of the minor allele was protective against ischemic stroke. Although only significant prior to adjustment for multiple testing, p.T101T (rs3815188; Exon 3) was associated with an increased risk of small-vessel stroke (OR: 1.56, P=0.008) and p.P380P (rs61749020; Exon 7) was associated with decreased risk of large-vessel stroke (OR: 0.35, P=0.047) in Caucasians. No significant associations were observed in the small African American series.

**Conclusion:**

Cysteine-affecting *NOTCH3* mutations are rare in patients with typical ischemic stroke, however our observation that common *NOTCH3* variants may be associated with risk of ischemic stroke warrants further study.

## Introduction

One of the most successful approaches to the mapping of disease-related genes has been the identification of rare inherited forms. The classical linkage method employing large familial aggregates that display Mendelian patterns of stroke inheritance (dominant/recessive) has identified genes involved in monogenic forms of disease [1,2]. Cerebral autosomal-dominant arteriopathy with subcortical infarctions and leukoencephalopathy (CADASIL; OMIM #125310), is a rare form of small-vessel occlusive disease. In 1996, Joutel and colleagues reported the identification of the first genetic locus for stroke, with pathogenic mutations in the *NOTCH3* gene [OMIM*600276] observed to cause CADASIL [3].

Over 50 *NOTCH3* mutations have been recorded, and to date there does not appear to be a consistent genotype-phenotype correlation with CADASIL [4]. The clinical phenotype of CADASIL usually presents with ischemic stroke or transient ischemic attack, cognitive deficits, migraine with aura, or psychiatric disturbance. Ischemic stroke is by far the most common manifestation, occurring in up to 85% of patients [5]. The disease course of CADASIL is variable, even in the same family [6]. The age-at-onset can range from 30-94 years, with early-onset forms not necessarily predicting a more severe symptomatic progression, and disease duration range between 3 to 43 years [7]. There is no specific treatment for CADASIL with most therapies focused on symptomatic control, the mean age-at-death is estimated to be about 60 years [5]. Interestingly, a *NOTCH3* mutation was identified in an octogenarian sporadic patient with a minor stroke demonstrating the potential role of *NOTCH3* mutations with less severe presentation [8,9]. Recently a novel mutation was reported p.L1515P, which is hypothesized to hyperactivate the NOTCH3 receptor, suggesting alternative mechanisms of pathogenicity [10]. However the actual pathomechanism behind *NOTCH3* mutations that are characteristic of CADASIL and ischemic stroke remains unclear. NOTCH3 signaling has also been implicated in ischemic stroke and studies suggest that the NOTCH3 protein is a determinant of stroke burden via vascular smooth muscle cells [11].

Given the reduced penetrance and clinical heterogeneity, we hypothesized that *NOTCH3* variants may play a greater role in ischemic stroke than previously thought. Herein, we present a complete exon screening of *NOTCH3* in 269 Caucasian probands with familial ischemic stroke and follow-up the identified variants in an association approach with a Caucasian and African-American patient-control series.

## Subjects and Methods

We utilized two different ischemic stroke patient-control series to investigate the role of *NOTCH3* variation in ischemic stroke; patient characteristics for each series are displayed in Table 1. For comprehensive sequence analysis, we employed a series of 269 Caucasian US familial stroke patients collected through the Siblings With Ischemic Stroke Study (SWISS). In brief, adult (>18 years old) probands were recruited at 70 US and Canadian medical centers with a study neurologist–confirmed ischemic stroke. Stroke was defined as rapidly developing signs of a focal or global disturbance of cerebral function, with symptoms lasting at least 24 hours or leading to death, with no apparent cause other than vascular origin (World Health Organization definition) [12]. Stroke was referred to as ischemic when computed tomography or magnetic resonance imaging of the brain was performed within 7 days of onset of stroke symptoms and identified the symptomatic cerebral infarct or failed to identify an alternative cause of symptoms. Probands were required to have reported at least 1 living full sibling with a history of stroke. No proband was enrolled with iatrogenic vasospastic or vasculitic stroke or if the stroke occurred in the setting of a mechanical heart valve or in the setting of untreated or actively treated bacterial endocarditis. Probands were also excluded if they were known to have CADASIL, Fabry disease, homocysteinuria, MELAS, or sickle-cell anemia. Study neurologists at each center assigned to the qualifying ischemic stroke of each proband a Trial of Org 10172 in Acute Stroke Treatment (TOAST) subtype diagnosis [13]. In addition, 95 healthy controls were obtained from Coriell Cell Repositories (Camden, NJ) for sequencing. These samples originated from different regions of the world (USA, Israel, Uruguay, Poland, Greece, Ireland, Australia, United Kingdom, Germany, Wales, Netherlands, Canada, Colombia and Cuba) noted to have an absence of neurological conditions by sample submitters [14]. This cohort of 95 healthy controls had a mean age of 78 years (range 70 to 95 years) and included 46 females and 49 males.

**Table 1:** Patient characteristics for each series.

	Familial Caucasian series		ISGS Caucasian series			ISGS African American series	
Variable	SWISS patients (N=269)	Controls (N=654)	Stroke patients (N=452)		Controls (N=350)	Stroke patients (N=167)	ISGS Controls (N=131)
Age	75 ± (36-99)	72 ± (23-96)	74 ± (31-103)		70 ± (30-100)	64 ± (28-101)	62 ± (30-98)
Gender (Male)	142 (55%)	268 (41%)	266 (59%)		176 (50%)	82 (49%)	57 (44%)
Age at stroke	67 ± (27-92)	N/A	66 ± (22-94)		N/A	56 ± (19-92)	N/A
Atrial fibrillation	29 (11%)	N/A	33 (7%)		8 (2%)	8 (5%)	0 (0%)
Coronary artery disease	37 (14%)	N/A	123 (27%)		38 (11%)	18 (11%)	7 (5%)
Diabetes	59 (23%)	N/A	105 (23%)		38 (11%)	51 (31%)	30 (23%)
Hypertension	181 (70%)	N/A	294 (65%)		129 (37%)	134 (80%)	58 (44%)
Current smoking	45 (17%)	N/A	100 (22%)		30 (9%)	62 (37%)	21 (16%)
**Type of stroke**							
Cardioembolic	32 (12%)	N/A	125 (28%)		N/A	29 (17%)	N/A
Large vessel	63 (24%)	N/A	90 (20%)		N/A	28 (17%)	N/A
Small vessel	80 (31%)	N/A	60 (13%)		N/A	46 (28%)	N/A
Other	15 (6%)	N/A	25 (6%)		N/A	2 (1%)	N/A
Undetermined	68 (26%)	N/A	152 (34%)		N/A	62 (37%)	N/A

The sample mean ± SD (minimum – maximum) is given for age and age at stroke. Information in the Familial Caucasian series was unavailable in stroke patients regarding atrial fibrillation (N=3), coronary artery disease (N=1), current smoking (N=2), and type of stroke (N=1), in all controls regarding atrial fibrillation, coronary artery disease, diabetes, hypertension, and current smoking, and in controls regarding age (N=2). In the ISGS Caucasian series, information was unavailable regarding atrial fibrillation (N=3), coronary artery disease (N=1), and hypertension (N=1). In the ISGS African American series, information was unavailable regarding atrial fibrillation (N=4), coronary artery disease (N=1). and hypertension (N=2). ISGS=Ischemic Stroke Genetics Study. SWISS=Siblings With Ischemic Stroke Study

For association and follow-up of variants, we supplemented the aforementioned 269 Caucasian familial stroke patients with a series of 654 Caucasian controls collected at the Mayo Clinic in Jacksonville, Florida, and we refer to this patient-control series as the familial Caucasian series. Additionally, we used a sporadic Caucasian patient-control series collected through the Ischemic Stroke Genetics Study (ISGS) which consisted of 452 stroke patients and 350 controls. Finally a small African American patient-control series collected as part of ISGS was studied, and this series was made of up 164 stroke patients and 131 controls. Subjects had age, gender, age at stroke, and type of stroke (cardioembolic, large vessel, small vessel, other, or undetermined) data collected. Additionally, information regarding atrial fibrillation, coronary artery disease, diabetes, hypertension, and current smoking was also collected in the two ISGS patient-control series’. We combined the familial Caucasian series and ISGS Caucasian series into a “combined Caucasian series” to be analyzed along with the individual series’. 

### Ethics Statement

The ethics committee and the institutional review board of the Mayo Clinic approved the study, and all participants provided written informed consent.

### Genetic analysis

For all 33 *NOTCH3* exons, bidirectional DNA sequencing was performed on an ABI 3730 DNA sequencer and analyzed using SeqScape v2.5 (Applied Biosystems). The variants were genotyped on a Sequenom MassArray iPLEX platform (San Diego, CA; primer sequences are available on request) and analyzed with Typer 4.0 software, an ABI on-demand Taqman assay (analyzed with SDS 2.2.2 software) or by direct exon sequencing on an ABI 3730. Assay Design software was used to multiplex the 34 variants identified during sequencing which were split into two iPLEX panels (primers available upon request). The rate of genotype calls was ≥95% in each series. Linkage disequilibrium (LD) measures were calculated and plotted using Haploview (Figure S1) [15].

### Statistical analysis

For *NOTCH3* variants occurring with a minor allele frequency of 1% or greater, associations with ischemic stroke were evaluated using logistic regression models, separately for the familial Caucasian series, ISGS Caucasian series, combined Caucasian series, and ISGS African American series. Odds ratios (ORs) and 95% confidence intervals (CIs) were estimated. Models involving the familial Caucasian series and combined Caucasian series were adjusted for age and gender, with adjustment also made for series in the combined Caucasian series. Models for the ISGS Caucasian and African American series’ were adjusted for age, gender, CAD, diabetes, hypertension, current smoking, and atrial fibrillation (ISGS Caucasian series only). As no ISGS African American controls had atrial fibrillation, this characteristic was not adjusted for in that series.

Associations of *NOTCH3* variants with ischemic stroke subtypes (cardioembolic stroke, large vessel stroke, small vessel stroke) were also evaluated in all individual series’ except the ISGS African American series due to its small sample size. In the primary analysis we utilized additive models (effect of each additional minor allele), though in secondary analysis we also examined dominant models (presence vs. absence of the minor allele). For *NOTCH3* variants with a minor allele frequency of less than 1%, we estimated the proportion of carriers in each series, separately for ischemic stroke patients and controls. We employed a single-step minP permutation correction in order to account for the number of statistical tests that were performed in our logistic regression analysis [16], separately for each series and separately for each ischemic stroke outcome (overall, cardioembolic, large-vessel, small-vessel). Following this multiple testing adjustment, p-values ≤0.0056 (familial Caucasian series), ≤0.0067 (ISGS Caucasian series), ≤0.0048 (combined Caucasian series), and ≤0.0061 (ISGS African American series) were considered as statistically significant. All statistical analyses were performed using R Statistical Software (version 2.14.0; R Foundation for Statistical Computing, Vienna, Austria).

## Results

We identified 39 variants in our comprehensive sequencing of the *NOTCH3* gene in the 269 familial ischemic stroke probands and 95 control subjects (Table 2). When examining the frequency of these variants utilizing both the familial Caucasian patient-control series and the ISGS Caucasian and African American patient-control series’, 26 variants were observed with a minor allele frequency of less than 1% in any of the individual series’; frequencies of these rare variants are detailed in Table 3 separately for stroke patients and controls in each series. Of note, in our stroke probands, we identified one novel variant in exon 4, which results in a histidine-to-arginine substitution p.H170R that is adjacent to known CADASIL mutants (p.R169C and p.G171C). This occurred in a 75-year-old Caucasian female with a TOAST subtype of ‘other determined etiology’. However when screened in controls this variant was observed at an equivalent frequency as in stroke patients in the combined Caucasian series (0.7% and 0.4% carriers, Table 3); p.H170R was not observed in the ISGS African American series.

**Table 2:** *NOTCH3* variants identified by Sequencing in SWISS probands and controls.

Position^1^	Exon	Genotype	SNP	Amino Acid
15303225	3	C>T	rs3815188	T101T
15302941	4	A>G	rs147373451	H170R
15302844	4	G>A	rs1043994	A202A
15302792	4	C>T	rs114457076	Y220Y
15302328	6	C>T	rs116239440	I315I
15300136	7	T>C	rs61749020	P380P
15299051	9	C>T	rs11670799	P496L
15299050	9	C>T	rs114207045	S497L
15298806	10	C>T	rs142762020	G498G
15298800	10	C>T	rs146055867	S500S
15298084	11	C>T	rs75068032	R558C
15298034	11	G>A	rs79926127	T575T
15297974	11	C>T	rs35793356	G594G
15296164	14	C>T	rs140040122	A734A
15295134	16	T>C	rs1043996	C846C
15292437	17	G>A	rs1043997	P914P
15291825	18	C>T	rs143695196	H981Y^†^
15291576	19	G>C	rs35769976	A1020P
15291553	19	G>T	rs146829488	W1028L
15290265	21	C>T	rs140642726	D1124D
15290238	21	C>A	rs112197217	H1133Q
15290007	22	G>A	rs10408676	V1183M
15288695	24	C>A	rs78926093	G1348G^†^
15285052	25	G>A	rs1044006	P1521P
15284978	25	C>G	rs150037063	L1547V
15284938	25	G>C	rs78501403	R1560P
15281580	26	A>T	rs201167365	D1598V^†^
15281344	27	A>G	rs149222385	E1638E
15280969	28	G>A	rs143411026	G1710D
15276739	30	T>C	rs16980398	A1842A
15273337	32	G>A	rs115582213	V1952M
15272410	33	G>A	rs142007575	V2011I^†^
15272343	33	C>T	rs145859816	P2033L
15272218	33	C>T	rs114447350	P2074L
15272199	33	G>T	rs141231747	G2081V
15272001	33	G>A	rs1044008	A2146A
15271771	33	T>C	rs1044009	A2223V
15271684	33	G>A	rs61731975	S2251S
15271628	33	T>C	rs61731974	P2271P

^1^ Chromosomal positions are based on the February 2009 (GRCH37/hg19) genome assembly. SNP=single nucleotide polymorphism. ^†^ Only observed in sequencing of 95 control subjects and not in subsequent screenings. na=not available.

**Table 3:** Summary of variants with a minor allele frequency of less than 1%.

			Familial SWISS Caucasian series (269 patients, 654 controls)				ISGS Caucasian series (452 patients, 350 controls)			Combined Caucasian series (721 patients, 1004 controls)			ISGS African American series (167 patients, 131 controls)		
				No. (%) of carriers				No. (%) of carriers			No. (%) of carriers			No. (%) of carriers	
SNP	Amino Acid	MA	MAF	Patients	Controls	MAF		Patients	Controls	MAF	Patients	Controls	MAF	Patients	Controls
rs147373451	H170R	C	0.4%	1 (0.4%)	6 (0.9%)	0.2%		2 (0.5%)	1 (0.3%)	0.3%	3 (0.4%)	7 (0.7%)	0.0%	0 (0%)	0 (0%)
rs114457076	Y220Y	A	0.2%	2 (0.8%)	2 (0.3%)	<0.1%		1 (0.2%)	0 (0.0%)	0.2%	3 (0.4%)	2 (0.2%)	0.0%	0 (0%)	0 (0%)
rs116239440	I315I	A	0.2%	2 (0.8%)	1 (0.2%)	0.1%		2 (0.5%)	0 (0.0%)	0.2%	4 (0.6%)	1 (0.1%)	0.0%	0 (0%)	0 (0%)
rs11670799	P496L	T	+	+	+	+		+	+	+	+	+	0.2%	0 (0.0%)	1 (1.0%)
rs114207045	S497L	A	0.4%	3 (1.1%)	5 (0.8%)	0.4%		4 (0.9%)	3 (0.9%)	0.4%	7 (1.0%)	8 (0.8%)	0.9%	2 (1.2%)	3 (2.29%)
rs142762020	G498G	A	<0.1%	1 (0.4%)	0 (0.0%)	0.0%		0 (0.0%)	0 (0.0%)	<0.1%	1 (0.1%)	0 (0.0%)	0.0%	0 (0%)	0 (0%)
rs146055867	S500S	A	0.2%	1 (0.4%)	3 (0.5%)	0.1%		2 (0.5%)	0 (0.0%)	0.2%	3 (0.4%)	3 (0.3%)	0.0%	0 (0%)	0 (0%)
rs75068032	R558C	T	<0.1%	1 (0.4%)	0 (0.0%)	0.0%		0 (0.0%)	0 (0.0%)	<0.1%	1 (0.1%)	0 (0.0%)	0.0%	0 (0%)	0 (0%)
rs79926127	T575T	A	0.8%	6 (2.2%)	9 (1.4%)	0.5%		7 (1.6%)	1 (0.3%)	0.7%	13 (1.8%)	10 (1.1%)	0.6%	0 (0.0%)	3 (2.8%)
rs35793356	G594G	A	0.2%	2 (0.7%)	2 (0.3%)	<0.1%		1 (0.2%)	0 (0.0%)	0.2%	3 (0.4%)	2 (0.2%)	+	+	+
rs140040122	A734A	A	0.2%	1 (0.4%)	3 (0.5%)	0.1%		1 (0.2%)	1 (0.3%)	0.2%	2 (0.3%)	4 (0.4%)	0.0%	0 (0%)	0 (0%)
rs146829488	W1028L	A	<0.1%	1 (0.4%)	0 (0.0%)	0.0%		0 (0.0%)	0 (0.0%)	<0.1%	1 (0.1%)	0 (0.0%)	0.0%	0 (0%)	0 (0%)
rs140642726	D1124D	A	<0.1%	1 (0.4%)	0 (0.0%)	0.0%		0 (0.0%)	0 (0.0%)	<0.1%	1 (0.1%)	0 (0.0%)	0.0%	0 (0%)	0 (0%)
rs112197217	H1133Q	T	+	+	+	+		+	+	+	+	+	0.2%	1 (0.6%)	0 (0.0%)
rs10408676	V1183M	T	+	+	+	0.5%		3 (0.7%)	5 (1.4%)	+	+	+	+	+	+
rs150037063	L1547V	C	0.2%	1 (0.4%)	2 (0.3%)	0.2%		1 (0.2%)	2 (0.6%)	0.2%	2 (0.3%)	4 (0.4%)	0.0%	0 (0%)	0 (0%)
rs149222385	E1638E	C	<0.1%	1 (0.4%)	0 (0.0%)	<0.1%		1 (0.2%)	0 (0.0%)	<0.1%	2 (0.3%)	0 (0.0%)	0.0%	0 (0%)	0 (0%)
rs143411026	G1710D	T	<0.1%	1 (0.4%)	0 (0.0%)	<0.1%		1 (0.2%)	0 (0.0%)	<0.1%	2 (0.3%)	0 (0.0%)	0.0%	0 (0%)	0 (0%)
rs16980398	A1842A	G	+	+	+	0.7%		5 (1.1%)	6 (1.7%)	+	+	+	+	+	+
rs115582213	V1952M	T	+	+	+	+		+	+	+	>+	+	0.0%	0 (0.0%)	0 (0.0%)
rs145859816	P2033L	A	<0.1%	1 (0.4%)	0 (0.0%)	0.0%		0 (0.0%)	0 (0.0%)	<0.1%	1 (0.1%)	0 (0.0%)	0.0%	0 (0%)	0 (0%)
rs114447350	P2074L	T	0.3%	2 (0.7%)	3 (0.5%)	0.2%		2 (0.5%)	1 (0.4%)	0.3%	4 (0.6%)	4 (0.5%)	+	+	+
rs141231747	G2081V	A	<0.1%	1 (0.4%)	0 (0.0%)	0.0%		0 (0.0%)	0 (0.0%)	<0.1%	1 (0.1%)	0 (0.0%)	0.0%	0 (0%)	0 (0%)
rs1044008	A2146A	T	+	+	+	+		+	+	+	+	+	0.5%	1 (0.6%)	2 (1.5%)
rs61731975	S2251S	A	0.4%	3 (1.1%)	4 (0.6%)	0.2%		2 (0.5%)	1 (0.3%)	0.3%	5 (0.7%)	5 (0.5%)	+	+	+
rs61731974	P2271P	G	0.2%	1 (0.4%)	3 (0.5%)	0.0%		0 (0.0%)	0 (0.0%)	0.1%	1 (0.1%)	3 (0.3%)	+	+	+

+ indicates that the SNP was observed with a minor allele frequency of 1% or greater in the given series. — indicates that the SNP was not observed in the given series. SNP=single nucleotide polymorphism. MA=minor allele. MAF=minor allele frequency.

**Figure 1:**
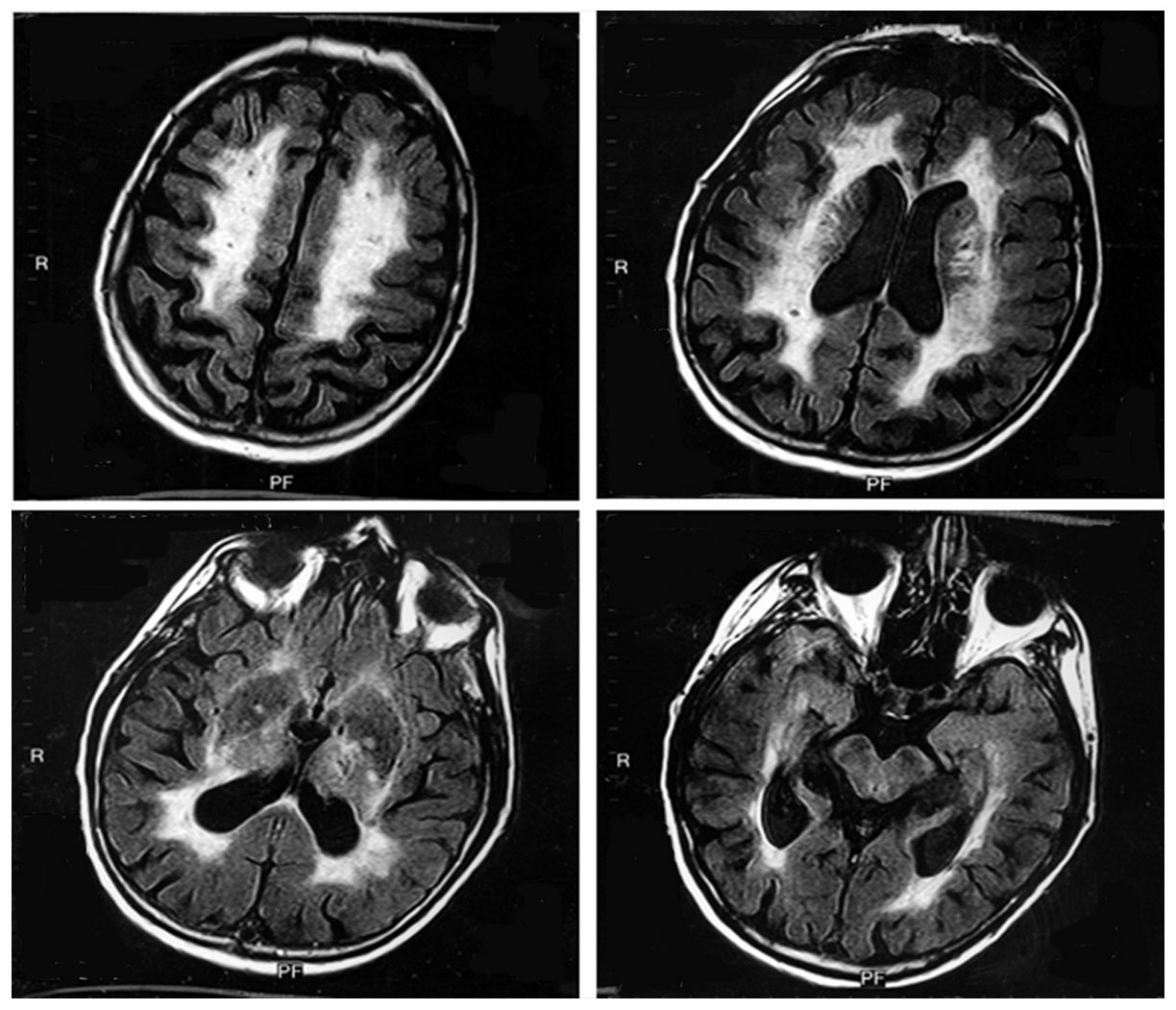
Bilateral small chronic infarcts and patchy and confluent areas of leukoaraiosis seen on fluid attenuated inversion recovery (FLAIR) MRI on patient with NOTCH3 p.R558C mutation.

One proband was observed to carry a known CADASIL causing mutation p.R558C (c. 1750 C>T). This patient was a 65-year-old man who presented for emergent medical attention with right arm weakness and mild dysarthria, which was caused by an acute infarct involving the posterior limb of the left internal capusle. The infarct occurred despite being prescribed cardizem, cardura, pravachol and aspirin. He had a history of one prior ischemic stroke 12 years earlier, sleep apnea, hyperlipidemia and hypertension, but not migraine headaches. MRI revealed a focal area of restricted diffusion corresponding to the presenting deficit along with bilateral small chronic infarcts and patchy leukoaraiosis (Figure 1). The affected sibling of the proband was also found to harbor the NOTCH3 p.R558C substitution.

A total of 19 variants were observed with a minor allele frequency of 1% or greater in any of the individual series (9 variants had a minor allele frequency ≥1% in all 4 series’); an evaluation of association of these 19 common variants with risk of overall ischemic in each series is shown in Table 4 under an additive model. In the combined Caucasian series, the only common *NOTCH3* variant that was significantly associated with ischemic stroke after correction for multiple testing was p.R1560P (rs78501403, OR: 0.50, 95% CI: 0.31-0.79, P=0.0022). This association was strongest in the familial Caucasian patient-control series (OR: 0.23, 95% CI: 0.10-0.55, P<0.001), whereas although a protective effect was observed in the ISGS Caucasian series, this did not approach significance (OR: 0.83, 95% CI: 0.41-1.66, P=0.60). Although not significant, noteworthy trends toward association with ischemic stroke to were observed for p.T101T (rs3815188) in the combined Caucasian series (OR: 1.22, 95% CI: 0.99-1.50, P=0.058), p.P2074L (rs114447350) in the ISGS African American series (OR: 2.29, 95% CI: 0.98-5.34, P=0.056), and p.P2271P (rs61731974) in the ISGS African American series (OR: 2.44, 95% CI: 0.91-6.54, P=0.077). Results of ischemic stroke association analysis were similar under a dominant model (Table S1).

**Table 4:** Single SNP associations with ischemic stroke under an additive model.

			Familial SWISS Caucasian series (269 patients, 654 controls)			ISGS Caucasian series (452 patients, 350 controls)			Combined Caucasian series (721 patients, 1004 controls)			ISGS African American series (167 patients, 131 controls)		
SNP	Amino Acid	MA	MAF	OR (95% CI)	P-value	MAF	OR (95% CI)	P-value	MAF	OR (95% CI)	P-value	MAF	OR (95% CI)	P-value
rs3815188	T101T	A	16.8%	1.40 (1.07, 1.84)	0.015	13.6%	1.03 (0.74, 1.44)	0.86	15.3%	1.22 (0.99, 1.50)	0.058	27.1%	1.05 (0.70, 1.56)	0.83
rs1043994	A202A	T	12.7%	0.91 (0.67, 1.22)	0.52	12.4%	0.93 (0.67, 1.29)	0.68	12.6%	0.95 (0.77, 1.17)	0.62	9.6%	0.84 (0.47, 1.51)	0.56
rs61749020	P380P	G	3.4%	0.56 (0.29, 1.08)	0.082	3.2%	1.17 (0.62, 2.19)	0.63	3.3%	0.85 (0.57, 1.28)	0.44	2.2%	1.63 (0.45, 5.91)	0.46
rs11670799	P496L	T	1.7%	0.32 (0.11, 0.92)	0.035	1.3%	1.70 (0.45, 6.42)	0.43	1.5%	0.87 (0.47, 1.62)	0.66	+	+	+
rs35793356	G594G	A	+	+	+	+	+	+	+	+	+	2.7%	2.66 (0.70, 10.08)	0.15
rs1043996	C846C	G^1^	31.7%	1.12 (0.91, 1.39)	0.29	27.4%	1.00 (0.78, 1.28)	1.00	29.7%	1.08 (0.92, 1.26)	0.35	26.4%	1.00 (0.67, 1.50)	0.99
rs1043997	P914P	T	15.0%	0.86 (0.65, 1.14)	0.29	13.3%	1.00 (0.72, 1.38)	0.99	14.2%	0.94 (0.77, 1.15)	0.56	36.8%	1.06 (0.73, 1.53)	0.77
rs35769976	A1020P	G	2.2%	0.84 (0.44, 1.62)	0.61	1.0%	1.33 (0.46, 3.79)	0.60	1.7%	0.88 (0.51, 1.51)	0.64	28.5%	1.12 (0.75, 1.67)	0.59
rs112197217	H1133Q	T	1.1%	1.35 (0.52, 3.48)	0.54	2.1%	0.88 (0.43, 1.82)	0.74	1.6%	0.99 (0.56, 1.74)	0.97	+	+	+
rs10408676	V1183M	T	1.6%	0.60 (0.25, 1.45)	0.26	+	+	+	1.1%	0.59 (0.28, 1.26)	0.17	23.7%	0.90 (0.59, 1.38)	0.63
rs1044006	P1521P	T	9.9%	0.81 (0.58, 1.15)	0.24	9.5%	1.03 (0.71, 1.50)	0.87	9.7%	0.97 (0.76, 1.23)	0.81	2.2%	0.86 (0.26, 2.79)	0.80
rs78501403	R1560P	G	3.7%	0.23 (0.10, 0.55)	<0.001	2.9%	0.83 (0.41, 1.66)	0.60	3.3%	0.50 (0.31, 0.79)	0.0022	4.4%	1.35 (0.54, 3.37)	0.52
rs16980398	A1842A	G	2.1%	0.76 (0.38, 1.52)	0.44	+	+	+	1.4%	0.76 (0.42, 1.38)	0.37	36.8%	1.09 (0.75, 1.59)	0.65
rs115582213	V1952M	T	1.3%	0.60 (0.22, 1.63)	0.32	1.1%	0.96 (0.34, 2.71)	0.93	1.2%	0.85 (0.44, 1.65)	0.64	+	+	+
rs114447350	P2074L	T	+	+	+	+	+	+	+	+	+	8.3%	2.29 (0.98, 5.34)	0.056
rs1044008	A2146A	T	4.6%	1.29 (0.81, 2.06)	0.28	4.4%	1.03 (0.63, 1.69)	0.90	4.5%	1.15 (0.83, 1.60)	0.41	+	+	+
rs1044009	A2223V	C	23.7%	1.22 (0.96, 1.54)	0.10	21.9%	0.97 (0.73, 1.29)	0.84	22.9%	1.16 (0.97, 1.38)	0.11	45.8%	1.05 (0.70, 1.56)	0.82
rs61731975	S2251S	A	+	+	+	+	+	+	+	+	+	8.7%	1.35 (0.68, 2.66)	0.39
rs61731974	P2271P	G	+	+	+	—	—	—	+	+	+	3.9%	2.44 (0.91, 6.54)	0.077

^1^ The minor allele for rs1043996 was G in the Caucasian series’ and A in the ISGS African American series. + indicates that the SNP was observed with a minor allele frequency of less than 1% or greater in the given series. --- indicates that the SNP was not observed in the given series. ORs and p-values result from logistic regression models adjusted for age and gender (Familial Caucasian series), age, gender, atrial fibrillation, coronary artery disease, diabetes, hypertension, and current smoking (ISGS Caucasian series), age, gender, and series (combined Caucasian series), and age, gender, coronary artery disease, diabetes, hypertension, and current smoking (ISGS African American series). ORs correspond to an additional minor allele. SNP=single nucleotide polymorphism. MA=minor allele. MAF=minor allele frequency. OR=odds ratio. CI=confidence interval. ISGS=Ischemic Stroke Genetics Study.

Associations of *NOTCH3* variants with cardioembolic, large-vessel, and small-vessel ischemic stroke in the combined Caucasian series under an additive model are displayed in Table 5. No variants were significantly associated with these ischemic stroke subtypes after multiple testing adjustment, although non-significant trends toward association were observed for p.T101T, which was associated with increased risk of small-vessel stroke (OR: 1.56, 95% CI: 1.12-2.18, P=0.008), and p.P380P (rs61749020), which was associated with a decreased risk of large-vessel stroke (OR: 0.35, 95% CI: 0.12-0.98, P=0.047). The aforementioned association with ischemic stroke for p.R1560P was relatively consistent in magnitude across ischemic stroke subtypes, though strongest for small-vessel stroke (OR: 0.36, P=0.053), followed by cardioembolic stroke (OR: 0.48, P=0.13) and large-vessel stroke (OR: 0.67, P=0.30). Results were similar under a dominant model (data not shown). Associations of *NOTCH3* variants with stroke subtypes in the individual familial Caucasian and ISGS Caucasian series’ are displayed in Tables S2 and S3, while genotype frequencies for each *NOTCH3* variant are shown in Tables S4-S7 for each series.

**Table 5:** Single SNP associations with ischemic stroke subtypes in the combined Caucasian series under an additive model.

				Association with cardioembolic stroke (157 patients, 1004 controls)			Association with large vessel stroke (153 patients, 1004 controls)		Association with small vessel stroke (140 patients, 1004 controls)	
SNP	Amino Acid	MA	MAF		OR (95% CI)	P-value	OR (95% CI)	P-value	OR (95% CI)	P-value
rs3815188	T101T	A	15.3%		0.96 (0.65, 1.43)	0.85	0.96 (0.66, 1.39)	0.83	1.56 (1.12, 2.18)	0.0082
rs1043994	A202A	T	12.6%		1.28 (0.91, 1.81)	0.16	1.05 (0.74, 1.49)	0.78	0.82 (0.56, 1.20)	0.31
rs61749020	P380P	G	3.3%		0.60 (0.25, 1.48)	0.27	0.35 (0.12, 0.98)	0.047	1.16 (0.59, 2.28)	0.67
rs11670799	P496L	T	1.5%		0.56 (0.12, 2.59)	0.46	0.67 (0.19, 2.29)	0.52	0.87 (0.29, 2.56)	0.79
rs1043996	C846C	G	29.7%		1.11 (0.84, 1.47)	0.48	1.02 (0.78, 1.34)	0.86	1.15 (0.88, 1.50)	0.31
rs1043997	P914P	T	14.2%		1.21 (0.86, 1.70)	0.28	1.08 (0.77, 1.51)	0.65	0.79 (0.54, 1.14)	0.20
rs35769976	A1020P	G	1.7%		0.53 (0.13, 2.23)	0.39	0.61 (0.20, 1.90)	0.40	1.07 (0.47, 2.43)	0.87
rs112197217	H1133Q	T	1.6%		1.07 (0.45, 2.52)	0.88	1.00 (0.38, 2.61)	1.00	1.22 (0.47, 3.17)	0.68
rs10408676	V1183M	T	1.1%		0.41 (0.06, 3.07)	0.39	0.30 (0.04, 2.17)	0.24	1.02 (0.37, 2.83)	0.97
rs1044006	P1521P	T	9.7%		1.25 (0.84, 1.86)	0.26	1.09 (0.74, 1.62)	0.66	0.81 (0.52, 1.25)	0.34
rs78501403	R1560P	G	3.3%		0.48 (0.18, 1.24)	0.13	0.67 (0.31, 1.43)	0.30	0.36 (0.13, 1.01)	0.053
rs16980398	A1842A	G	1.4%		0.35 (0.05, 2.53)	0.30	0.74 (0.25, 2.19)	0.59	1.22 (0.55, 2.70)	0.62
rs115582213	V1952M	T	1.2%		1.75 (0.66, 4.65)	0.26	1.21 (0.44, 3.27)	0.71	0.49 (0.11, 2.09)	0.33
rs1044008	A2146A	T	4.5%		1.11 (0.63, 1.94)	0.72	0.67 (0.33, 1.34)	0.25	1.28 (0.75, 2.17)	0.37
rs1044009	A2223V	C	22.9%		1.22 (0.90, 1.65)	0.21	1.08 (0.80, 1.45)	0.62	1.25 (0.93, 1.66)	0.14

ORs and p-values result from logistic regression models adjusted for age, gender, and series. ORs correspond to an additional minor allele. SNP=single nucleotide polymorphism. MA=minor allele. MAF=minor allele frequency. OR=odds ratio. CI=confidence interval.

## Discussion

Rare mutations within the *NOTCH3* gene resulting in the gain or loss of a cysteine residue produce the CADASIL phenotype. We set out to examine whether CADASIL-linked mutations can also produce a clinical phenotype that is more reminiscent of typical ischemic stroke. In addition, we examined if other coding variants in *NOTCH3*, common or rare variation, affect the individual susceptibility to ischemic stroke.

One of the variants identified is a known pathogenic CADASIL substitution (p.R558C) and was identified in a proband with a history of small vessel stroke. In addition to p.R558C substitution, we observed a number of rare variants within the *NOTCH3* gene, 15 of which are non-synonymous. When evaluating associations with ischemic stroke for common variants, we observed an association for p.R1560P in the combined Caucasian series that withstood correction for multiple testing, where presence of the minor allele was associated with a lower risk of ischemic stroke with an odds ratio of 0.50. This protective effect for p.R1560P was also observed when considering ischemic stroke subtypes (although not statistically significant in these lower-powered analyses), and was strongest for small-vessel ischemic stroke. Employing the Polyphen (Polymorphism phenotyping) freeware, which predicts the effect of an amino acid substitution, suggests that the p.R1560P may possibly damage the structure and function of the NOTCH3 protein and could support a functional effect by this substitution [17].

A recent study by Schmidt and colleagues examined the presence of *NOTCH3* variants in an elderly series and assessed whether they play a role in age-related small vessel disease [18]. They observed a risk association of a number of common variants and the presence of white matter lesions; however the association was only present in hypertensive subjects. Interestingly, although they sequenced almost 300 individuals, they did not observe the p.R1560P variant, suggesting that the p.R1560P variant may display different allele frequencies across populations. In addition the p.R1560P substitution was not observed in >4000 individuals in the exome variant server database. In the current study all carriers were Sanger sequenced to confirm the presence and the publicly available data may suggest that this exon is poorly captured through exome approaches. Indeed, to further investigate we attempted to impute this variant in 11 datasets ranging in size from 900 participants to over 5000 participants. The SNP of interest appeared monomorphic in all imputed datasets except two cohorts where the minor allele frequency in both was less than 0.5%. However, in all imputed datasets, imputation quality (RSQ) was much less than the gold standard cut-off of 0.30, with RSQ metrics ranging from 0 to a high of 0.038. It is known that imputation generally functions less accurately for rarer variants. All imputed datasets were generated using MACH and miniMac and default settings for imputation [http://genome.sph.umich.edu/wiki/Minimac] with the 1000 Genomes Project phase 1 alpha freeze version 3 vcf file (multi-ethnic panel) as the reference for imputation [http://www.1000genomes.org]. Therefore further specific genotyping of this variant may be warranted.

There are several limitations of our study that should be acknowledged. Chief among these is the relatively small sample size. As a result, power to detect associations of *NOTCH3* variants with ischemic stroke and ischemic stroke subtypes is low, and the possibility of Type II error (i.e. a false-negative association) is important to acknowledge, especially after correction for multiple testing. This is particularly true in the African American series and in examination of associations of *NOTCH3* variants with ischemic stroke subtypes. Related to this, assessment of rare variants is challenging in our relatively small study. Also, though the finding regarding p.R1560P achieved statistical significance after multiple testing adjustment, given the stronger association in the Familial Caucasian series than in the ISGS Caucasian series, it will be important to validate this finding as well as other results of our study involving both common and rare variants in larger series’. 

The NOTCH pathway is a fundamental signaling mechanism determining cell fate choices [19]. NOTCH 1-4 are cell surface receptors, which interact with membrane-bound ligands transducing signals between neighboring cells. The NOTCH3 receptor has been reported to promote vascular smooth muscle cell survival. To date the confirmed pathogenic CADASIL mutations are located in the epidermal growth factor (EGF)-like repeat domains at the extracellular N-domain of the receptor. Missense and splice-site mutations and in-frame deletions have been observed in patients. Mutations appear to affect highly conserved cysteine residues. Within each wild-type EGF-like repeat there are six cysteine residues, while in CADASIL mutation patients there is a loss or gain of a cysteine residue. This uneven number of cysteine residues has been hypothesized to effect differential protein interactions and the possible multimerization of mutant NOTCH3.

The association of *NOTCH3* p.R1560P suggests that disruption of the normal NOTCH3 receptor function could modulate the risk of ischemic stroke. Additional studies in large patient-control series and other ethnicities are required to fully elucidate the role of *NOTCH3* variation in disease and the underlying pathomechanism that results in ischemia.

## Supporting Information

Figure S1 **Linkage disequilibrium (LD) plots for common *NOTCH3* variants.**
(PPTX)

Table S1 **Single SNP associations with ischemic stroke under a dominant model.**
(DOCX)

Table S2 **Single SNP associations with ischemic stroke subtypes in the familial Caucasian series under an additive model.**
(DOCX)

Table S3 **Single SNP associations with ischemic stroke subtypes in the ISGS Caucasian series under an additive model.**
(DOCX)

Table S4 **Genotype frequencies in the Familial Caucasian series.**
(DOCX)

Table S5 **Genotype frequencies in the ISGS Caucasian series.**
(DOCX)

Table S6 **Genotype frequencies in the combined Caucasian series.**
(DOCX)

Table S7 **Genotype frequencies in the ISGS African American series.**
(DOCX)
