# Supplementary material for: NOTCH3 Variants and Risk of Ischemic Stroke
Source: PLoS One. 2013 Sep 23;8(9):e75035. doi: 10.1371/journal.pone.0075035 (PMC3781028; doi:10.1371/journal.pone.0075035)
Supplement: Figure S1 — Linkage disequilibrium (LD) plots for common NOTCH3 variants. (PPTX) [file pone.0075035.s001.pptx]

## Slide 1
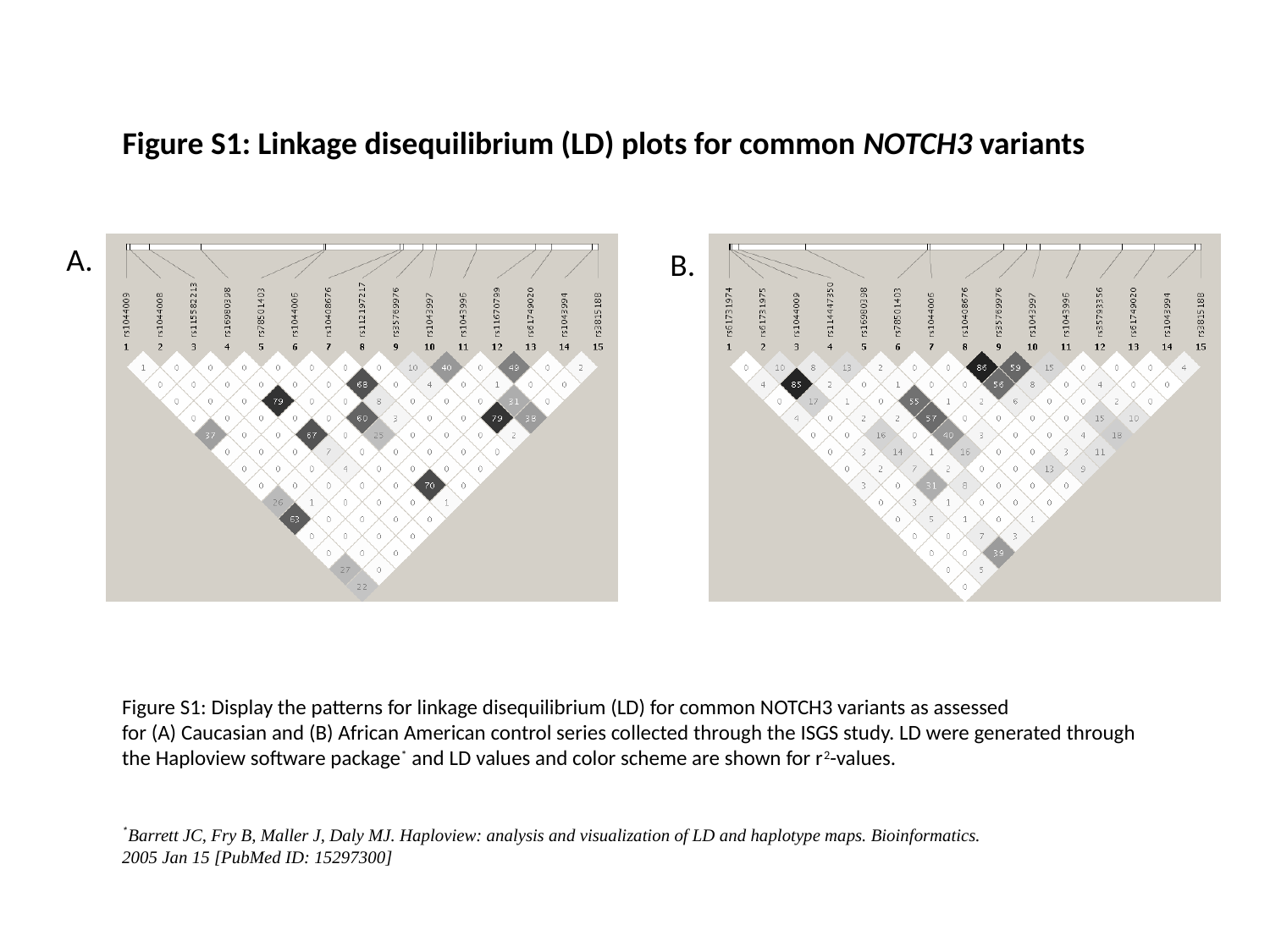

Figure S1: Linkage disequilibrium (LD) plots for common NOTCH3 variants
A.
B.
Figure S1: Display the patterns for linkage disequilibrium (LD) for common NOTCH3 variants as assessed
for (A) Caucasian and (B) African American control series collected through the ISGS study. LD were generated through
the Haploview software package* and LD values and color scheme are shown for r2-values.
*Barrett JC, Fry B, Maller J, Daly MJ. Haploview: analysis and visualization of LD and haplotype maps. Bioinformatics.
2005 Jan 15 [PubMed ID: 15297300]
