# Supplementary material for: NOTCH3 Variants and Risk of Ischemic Stroke
Source: PLoS One. 2013 Sep 23;8(9):e75035. doi: 10.1371/journal.pone.0075035 (PMC3781028; doi:10.1371/journal.pone.0075035)
Supplement: Table S1 — Single SNP associations with ischemic stroke under a dominant model. (DOCX) [file pone.0075035.s002.docx]

**Table S1: Single SNP associations with ischemic stroke under a dominant model**

|  |  |  | Familial Caucasian series (269 patients, 654 controls) | | | ISGS Caucasian series (452 patients, 350 controls) | | | Combined Caucasian series (721 patients, 1004 controls) | | | ISGS African American series (167 patients, 131 controls) | | |
| --- | --- | --- | --- | --- | --- | --- | --- | --- | --- | --- | --- | --- | --- | --- |
| SNP | Amino Acid | MA | MAF | OR (95% CI) | P-value | MAF | OR (95% CI) | P-value | MAF | OR (95% CI) | P-value | MAF | OR (95% CI) | P-value |
| rs3815188 | T101T | A | 16.8% | 1.33 (0.98, 1.81) | 0.070 | 13.6% | 1.03 (0.72, 1.45) | 0.89 | 15.3% | 1.19 (0.95, 1.49) | 0.13 | 27.1% | 1.28 (0.76, 2.16) | 0.35 |
| rs1043994 | A202A | T | 12.7% | 0.95 (0.67, 1.35) | 0.78 | 12.4% | 0.93 (0.64, 1.34) | 0.68 | 12.6% | 0.98 (0.77, 1.25) | 0.89 | 9.6% | 0.95 (0.49, 1.84) | 0.88 |
| rs61749020 | P380P | G | 3.4% | 0.56 (0.29, 1.08) | 0.082 | 3.2% | 1.15 (0.60, 2.18) | 0.68 | 3.3% | 0.83 (0.55, 1.26) | 0.46 | 2.2% | 1.63 (0.45, 5.91) | 0.46 |
| rs11670799 | P496L | T | 1.7% | 0.32 (0.11, 0.92) | 0.035 | 1.3% | 1.70 (0.45, 6.42) | 0.43 | 1.5% | 0.87 (0.47, 1.62) | 0.66 | + | + | + |
| rs35793356 | G594G | A | + | + | + | + | + | + | + | + | + | 2.7% | 2.66 (0.70, 10.08) | 0.15 |
| rs1043996 | C846C | G^1^ | 31.7% | 1.09 (0.82, 1.47) | 0.54 | 27.4% | 0.97 (0.71, 1.32) | 0.85 | 29.7% | 1.08 (0.88, 1.32) | 0.47 | 26.4% | 1.23 (0.73, 2.07) | 0.44 |
| rs1043997 | P914P | T | 15.0% | 0.89 (0.64, 1.23) | 0.47 | 13.3% | 1.01 (0.70, 1.44) | 0.97 | 14.2% | 0.97 (0.77, 1.23) | 0.82 | 36.8% | 1.16 (0.69, 1.96) | 0.58 |
| rs35769976 | A1020P | G | 2.2% | 0.95 (0.46, 1.96) | 0.89 | 1.0% | 1.33 (0.46, 3.79) | 0.6 | 1.7% | 0.96 (0.53, 1.72) | 0.88 | 28.5% | 1.20 (0.71, 2.00) | 0.50 |
| rs112197217 | H1133Q | T | 1.1% | 1.35 (0.52, 3.48) | 0.54 | 2.1% | 0.96 (0.45, 2.06) | 0.92 | 1.6% | 1.04 (0.58, 1.85) | 0.9 | + | + | + |
| rs10408676 | V1183M | T | 1.6% | 0.62 (0.25, 1.57) | 0.31 | + | + | + | 1.1% | 0.60 (0.28, 1.32) | 0.21 | 23.7% | 0.99 (0.59, 1.68) | 0.98 |
| rs1044006 | P1521P | T | 9.9% | 0.87 (0.60, 1.28) | 0.49 | 9.5% | 0.97 (0.65, 1.46) | 0.90 | 9.7% | 1.00 (0.77, 1.31) | 0.97 | 2.2% | 0.86 (0.26, 2.79) | 0.80 |
| rs78501403 | R1560P | G | 3.7% | 0.23 (0.10, 0.55) | 0.0009 | 2.9% | 0.83 (0.41, 1.66) | 0.60 | 3.3% | 0.50 (0.31, 0.79) | 0.0032 | 4.4% | 1.28 (0.49, 3.38) | 0.62 |
| rs16980398 | A1842A | G | 2.1% | 0.87 (0.40, 1.92) | 0.73 | + | + | + | 1.4% | 0.83 (0.43, 1.62) | 0.59 | 36.8% | 1.51 (0.90, 2.56) | 0.12 |
| rs115582213 | V1952M | T | 1.3% | 0.60 (0.22, 1.63) | 0.32 | 1.1% | 0.96 (0.34, 2.71) | 0.93 | 1.2% | 0.85 (0.44, 1.65) | 0.64 | + | + | + |
| rs114447350 | P2074L | T | + | + | + | + | + | + | + | + | + | 8.3% | 2.29 (0.98, 5.34) | 0.056 |
| rs1044008 | A2146A | T | 4.6% | 1.30 (0.80, 2.11) | 0.29 | 4.4% | 1.06 (0.60, 1.87) | 0.84 | 4.5% | 1.18 (0.82, 1.69) | 0.38 | + | + | + |
| rs1044009 | A2223V | C | 23.7% | 1.24 (0.92, 1.66) | 0.16 | 21.9% | 1.01 (0.72, 1.42) | 0.94 | 22.9% | 1.22 (0.99, 1.52) | 0.066 | 45.8% | 1.00 (0.53, 1.89) | 0.99 |
| rs61731975 | S2251S | A | + | + | + | + | + | + | + | + | + | 8.7% | 1.41 (0.69, 2.89) | 0.35 |
| rs61731974 | P2271P | G | + | + | + | --- | --- | --- | + | + | + | 3.9% | 3.15 (1.02, 9.75) | 0.046 |
| ^1^ The minor allele for rs1043996 was G in the Caucasian series’ and A in the ISGS African American series. + indicates that the SNP was observed with a minor allele frequency of less than 1% or greater in the given series. --- indicates that the SNP was not observed in the given series. ORs and p-values result from logistic regression models adjusted for age and gender (Familial Caucasian series), age, gender, atrial fibrillation, coronary artery disease, diabetes, hypertension, and current smoking (ISGS Caucasian series), age, gender, and series (combined Caucasian series), and age, gender, coronary artery disease, diabetes, hypertension, and current smoking (ISGS African American series). ORs correspond to presence of the minor allele. SNP=single nucleotide polymorphism. MA=minor allele. MAF=minor allele frequency. OR=odds ratio. CI=confidence interval. ISGS=Ischemic Stroke Genetics Study. | | | | | | | | | | | | | | |
