# Supplementary material for: NOTCH3 Variants and Risk of Ischemic Stroke
Source: PLoS One. 2013 Sep 23;8(9):e75035. doi: 10.1371/journal.pone.0075035 (PMC3781028; doi:10.1371/journal.pone.0075035)
Supplement: Table S2 — Single SNP associations with ischemic stroke subtypes in the familial Caucasian series under an additive model. (DOCX) [file pone.0075035.s003.docx]

**Table S2: Single SNP associations with ischemic stroke subtypes in the familial Caucasian series under an additive model**

|  |  |  |  | Association with cardioembolic stroke (32 patients, 654 controls) | | Association with large vessel stroke (63 patients, 654 controls) | | Association with small vessel stroke (80 patients, 654 controls) | |
| --- | --- | --- | --- | --- | --- | --- | --- | --- | --- |
| SNP | Amino Acid | MA | MAF | OR (95% CI) | P-value | OR (95% CI) | P-value | OR (95% CI) | P-value |
| rs3815188 | T101T | A | 16.8% | 1.22 (0.60, 2.5) | 0.59 | 1.20 (0.72, 1.99) | 0.49 | 2.00 (1.34, 3.00) | 0.0007 |
| rs1043994 | A202A | T | 12.7% | 1.05 (0.54, 2.02) | 0.89 | 1.26 (0.80, 1.98) | 0.32 | 0.54 (0.30, 0.95) | 0.034 |
| rs61749020 | P380P | G | 3.4% | N/A | N/A | 0.18 (0.02, 1.34) | 0.094 | 0.93 (0.38, 2.27) | 0.87 |
| rs11670799 | P496L | T | 1.7% | N/A | N/A | 0.32 (0.04, 2.45) | 0.27 | 0.51 (0.12, 2.21) | 0.37 |
| rs1043996 | C846C | G | 31.7% | 1.03 (0.61, 1.75) | 0.91 | 1.25 (0.86, 1.81) | 0.24 | 1.21 (0.86, 1.69) | 0.27 |
| rs1043997 | P914P | T | 15.0% | 0.79 (0.39, 1.59) | 0.51 | 1.17 (0.76, 1.81) | 0.47 | 0.61 (0.37, 1.02) | 0.057 |
| rs35769976 | A1020P | G | 2.2% | 0.62 (0.10, 4.01) | 0.62 | 0.34 (0.05, 2.26) | 0.26 | 1.33 (0.58, 3.02) | 0.50 |
| rs112197217 | H1133Q | T | 1.1% | N/A | N/A | 1.64 (0.35, 7.68) | 0.53 | 2.00 (0.54, 7.46) | 0.30 |
| rs10408676 | V1183M | T | 1.6% | 0.81 (0.12, 5.47) | 0.83 | N/A | N/A | 1.21 (0.43, 3.37) | 0.72 |
| rs1044006 | P1521P | T | 9.9% | 0.63 (0.26, 1.56) | 0.32 | 0.99 (0.57, 1.70) | 0.97 | 0.61 (0.33, 1.11) | 0.10 |
| rs78501403 | R1560P | G | 3.7% | N/A | N/A | 0.30 (0.07, 1.26) | 0.10 | 0.25 (0.06, 1.07) | 0.062 |
| rs16980398 | A1842A | G | 2.1% | 0.71 (0.12, 4.23) | 0.71 | 0.41 (0.07, 2.48) | 0.33 | 1.41 (0.64, 3.13) | 0.40 |
| rs115582213 | V1952M | T | 1.3% | 0.95 (0.12, 7.44) | 0.96 | 0.92 (0.21, 4.13) | 0.92 | N/A | N/A |
| rs1044008 | A2146A | T | 4.6% | 1.44 (0.51, 4.11) | 0.49 | 0.53 (0.16, 1.72) | 0.29 | 1.74 (0.91, 3.34) | 0.093 |
| rs1044009 | A2223V | C | 23.7% | 0.97 (0.54, 1.75) | 0.93 | 1.15 (0.76, 1.73) | 0.52 | 1.40 (0.98, 2.00) | 0.066 |
| ORs and p-values result from logistic regression models adjusted for age and gender. N/A indicates a SNP for which there were either no stroke patients (of the given subtype) or controls with a copy of the minor allele, making logistic regression analysis impossible. ORs correspond to an additional minor allele. SNP=single nucleotide polymorphism. MA=minor allele. MAF=minor allele frequency. OR=odds ratio. CI=confidence interval. | | | | | | | | | |
