# Supplementary material for: NOTCH3 Variants and Risk of Ischemic Stroke
Source: PLoS One. 2013 Sep 23;8(9):e75035. doi: 10.1371/journal.pone.0075035 (PMC3781028; doi:10.1371/journal.pone.0075035)
Supplement: Table S3 — Single SNP associations with ischemic stroke subtypes in the ISGS Caucasian series under an additive model. (DOCX) [file pone.0075035.s004.docx]

**Table S3: Single SNP associations with ischemic stroke subtypes in the ISGS Caucasian series under an additive model**

|  |  |  |  | Association with cardioembolic stroke (125 patients, 350 controls) | | Association with large vessel stroke (90 patients, 350 controls) | | Association with small vessel stroke (60 patients, 350 controls) | |
| --- | --- | --- | --- | --- | --- | --- | --- | --- | --- |
| SNP | Amino Acid | MA | MAF | OR (95% CI) | P-value | OR (95% CI) | P-value | OR (95% CI) | P-value |
| rs3815188 | T101T | A | 13.6% | 0.85 (0.50, 1.44) | 0.55 | 0.82 (0.45, 1.50) | 0.52 | 0.94 (0.48, 1.86) | 0.87 |
| rs1043994 | A202A | T | 12.4% | 1.23 (0.77, 1.97) | 0.38 | 0.59 (0.31, 1.12) | 0.11 | 1.24 (0.68, 2.23) | 0.48 |
| rs61749020 | P380P | G | 3.2% | 0.69 (0.22, 2.13) | 0.52 | 0.52 (0.13, 2.06) | 0.35 | 1.93 (0.59, 6.34) | 0.28 |
| rs11670799 | P496L | T | 1.3% | 0.68 (0.08, 6.03) | 0.73 | 0.96 (0.12, 7.79) | 0.97 | 1.65 (0.19, 14.09) | 0.65 |
| rs1043996 | C846C | G | 27.4% | 1.05 (0.72, 1.52) | 0.81 | 0.70 (0.44, 1.11) | 0.13 | 1.02 (0.63, 1.67) | 0.93 |
| rs1043997 | P914P | T | 13.3% | 1.28 (0.81, 2.04) | 0.29 | 0.71 (0.39, 1.29) | 0.27 | 1.07 (0.59, 1.97) | 0.82 |
| rs35769976 | A1020P | G | 1.0% | 0.56 (0.07, 4.71) | 0.59 | 1.60 (0.31, 8.25) | 0.57 | N/A | N/A |
| rs112197217 | H1133Q | T | 2.1% | 1.12 (0.42, 2.99) | 0.82 | 0.57 (0.15, 2.12) | 0.40 | 0.64 (0.14, 3.00) | 0.57 |
| rs1044006 | P1521P | T | 9.5% | 1.48 (0.87, 2.52) | 0.15 | 0.85 (0.43, 1.68) | 0.65 | 1.18 (0.57, 2.44) | 0.66 |
| rs78501403 | R1560P | G | 2.9% | 0.84 (0.27, 2.56) | 0.76 | 1.19 (0.37, 3.83) | 0.77 | 0.84 (0.15, 4.59) | 0.84 |
| rs115582213 | V1952M | T | 1.1% | 1.38 (0.37, 5.20) | 0.63 | 1.63 (0.37, 7.12) | 0.51 | 1.61 (0.28, 9.20) | 0.59 |
| rs1044008 | A2146A | T | 4.4% | 0.85 (0.40, 1.78) | 0.66 | 0.87 (0.34, 2.22) | 0.78 | 0.95 (0.33, 2.74) | 0.93 |
| rs1044009 | A2223V | C | 21.9% | 1.18 (0.78, 1.79) | 0.44 | 0.80 (0.49, 1.33) | 0.39 | 0.95 (0.54, 1.68) | 0.87 |
| ORs and p-values result from logistic regression models adjusted for age, gender, atrial fibrillation, coronary artery disease, diabetes, hypertension, and current smoking. N/A indicates a SNP for which there were either no stroke patients (of the given subtype) or controls with a copy of the minor allele, making logistic regression analysis impossible. ORs correspond to an additional minor allele. SNP=single nucleotide polymorphism. MA=minor allele. MAF=minor allele frequency. OR=odds ratio. CI=confidence interval. ISGS=Ischemic Stroke Genetics Study. | | | | | | | | | |
