# Supplementary material for: NOTCH3 Variants and Risk of Ischemic Stroke
Source: PLoS One. 2013 Sep 23;8(9):e75035. doi: 10.1371/journal.pone.0075035 (PMC3781028; doi:10.1371/journal.pone.0075035)
Supplement: Table S4 — Genotype frequencies in the Familial Caucasian series. (DOCX) [file pone.0075035.s005.docx]

**Table S4: Genotype frequencies in the Familial Caucasian series**

|  | Controls (N=654) | | | Stroke patients (N=269) | | |
| --- | --- | --- | --- | --- | --- | --- |
| SNP | Major/Major | Major/Minor | Minor/Minor | Major/Major | Major/Minor | Minor/Minor |
| rs3815188 | 454 (70.2%) | 185 (28.6%) | 8 (1.2%) | 174 (64.9%) | 82 (30.6%) | 12 (4.5%) |
| rs147373451 | 648 (99.1%) | 6 (0.9%) | 0 (0.0%) | 264 (99.6%) | 1 (0.4%) | 0 (0.0%) |
| rs1043994 | 499 (77.1%) | 128 (19.8%) | 20 (3.1%) | 209 (77.7%) | 55 (20.4%) | 5 (1.9%) |
| rs114457076 | 652 (99.7%) | 2 (0.3%) | 0 (0.0%) | 265 (99.3%) | 2 (0.7%) | 0 (0.0%) |
| rs116239440 | 653 (99.8%) | 1 (0.2%) | 0 (0.0%) | 265 (99.3%) | 2 (0.7%) | 0 (0.0%) |
| rs61749020 | 591 (92.2%) | 50 (7.8%) | 0 (0.0%) | 247 (95.4%) | 12 (4.6%) | 0 (0.0%) |
| rs11670799 | 599 (95.7%) | 27 (4.3%) | 0 (0.0%) | 265 (98.5%) | 4 (1.5%) | 0 (0.0%) |
| rs114207045 | 649 (99.2%) | 5 (0.8%) | 0 (0.0%) | 264 (98.9%) | 3 (1.1%) | 0 (0.0%) |
| rs142762020 | 648 (100%) | 0 (0.0%) | 0 (0.0%) | 268 (99.6%) | 1 (0.4%) | 0 (0.0%) |
| rs146055867 | 651 (99.5%) | 3 (0.5%) | 0 (0.0%) | 266 (99.6%) | 1 (0.4%) | 0 (0.0%) |
| ss153922421 | 634 (100%) | 0 (0.0%) | 0 (0.0%) | 268 (99.6%) | 1 (0.4%) | 0 (0.0%) |
| rs79926127 | 625 (98.6%) | 9 (1.4%) | 0 (0.0%) | 263 (97.8%) | 6 (2.2%) | 0 (0.0%) |
| rs35793356 | 646 (99.7%) | 2 (0.3%) | 0 (0.0%) | 267 (99.3%) | 2 (0.7%) | 0 (0.0%) |
| rs140040122 | 644 (99.5%) | 3 (0.5%) | 0 (0.0%) | 268 (99.6%) | 1 (0.4%) | 0 (0.0%) |
| rs1043996 | 309 (47.9%) | 273 (42.3%) | 63 (9.8%) | 122 (45.9%) | 109 (41%) | 35 (13.2%) |
| rs1043997 | 472 (72.6%) | 155 (23.8%) | 23 (3.5%) | 198 (74.4%) | 62 (23.3%) | 6 (2.3%) |
| rs35769976 | 627 (96%) | 22 (3.4%) | 4 (0.6%) | 256 (95.9%) | 11 (4.1%) | 0 (0.0%) |
| rs146829488 | 648 (100%) | 0 (0.0%) | 0 (0.0%) | 268 (99.6%) | 1 (0.4%) | 0 (0.0%) |
| rs140642726 | 647 (100%) | 0 (0.0%) | 0 (0.0%) | 268 (99.6%) | 1 (0.4%) | 0 (0.0%) |
| rs112197217 | 641 (98%) | 13 (2%) | 0 (0.0%) | 260 (97.4%) | 7 (2.6%) | 0 (0.0%) |
| rs10408676 | 633 (96.8%) | 19 (2.9%) | 2 (0.3%) | 261 (97.8%) | 6 (2.2%) | 0 (0.0%) |
| rs1044006 | 519 (81.2%) | 108 (16.9%) | 12 (1.9%) | 222 (82.8%) | 45 (16.8%) | 1 (0.4%) |
| rs150037063 | 652 (99.7%) | 2 (0.3%) | 0 (0.0%) | 266 (99.6%) | 1 (0.4%) | 0 (0.0%) |
| rs78501403 | 566 (90.4%) | 60 (9.6%) | 0 (0.0%) | 260 (97.7%) | 6 (2.3%) | 0 (0.0%) |
| rs149222385 | 653 (100%) | 0 (0.0%) | 0 (0.0%) | 266 (99.6%) | 1 (0.4%) | 0 (0.0%) |
| rs143411026 | 654 (100%) | 0 (0.0%) | 0 (0.0%) | 266 (99.6%) | 1 (0.4%) | 0 (0.0%) |
| rs16980398 | 630 (96.3%) | 19 (2.9%) | 5 (0.8%) | 258 (96.6%) | 9 (3.4%) | 0 (0.0%) |
| rs115582213 | 635 (97.1%) | 19 (2.9%) | 0 (0.0%) | 262 (98.1%) | 5 (1.9%) | 0 (0.0%) |
| rs145859816 | 648 (100%) | 0 (0.0%) | 0 (0.0%) | 268 (99.6%) | 1 (0.4%) | 0 (0.0%) |
| rs114447350 | 593 (99.5%) | 3 (0.5%) | 0 (0.0%) | 267 (99.3%) | 2 (0.7%) | 0 (0.0%) |
| rs141231747 | 644 (100%) | 0 (0.0%) | 0 (0.0%) | 265 (99.6%) | 1 (0.4%) | 0 (0.0%) |
| rs1044008 | 599 (91.6%) | 54 (8.3%) | 1 (0.2%) | 239 (89.5%) | 27 (10.1%) | 1 (0.4%) |
| rs1044009 | 372 (60.4%) | 209 (33.9%) | 35 (5.7%) | 150 (56%) | 96 (35.8%) | 22 (8.2%) |
| rs61731975 | 650 (99.4%) | 4 (0.6%) | 0 (0.0%) | 264 (98.9%) | 3 (1.1%) | 0 (0.0%) |
| rs61731974 | 651 (99.5%) | 3 (0.5%) | 0 (0.0%) | 266 (99.6%) | 1 (0.4%) | 0 (0.0%) |
