# Supplementary material for: NOTCH3 Variants and Risk of Ischemic Stroke
Source: PLoS One. 2013 Sep 23;8(9):e75035. doi: 10.1371/journal.pone.0075035 (PMC3781028; doi:10.1371/journal.pone.0075035)
Supplement: Table S5 — Genotype frequencies in the ISGS Caucasian series. (DOCX) [file pone.0075035.s006.docx]

**Table S5: Genotype frequencies in the ISGS Caucasian series**

|  | Controls (N=350) | | | Stroke patients (N=452) | | |
| --- | --- | --- | --- | --- | --- | --- |
| SNP | Major/Major | Major/Minor | Minor/Minor | Major/Major | Major/Minor | Minor/Minor |
| rs3815188 | 254 (74.3%) | 85 (24.9%) | 3 (0.9%) | 329 (72.9%) | 119 (26.4%) | 3 (0.7%) |
| rs147373451 | 348 (99.7%) | 1 (0.3%) | 0 (0.0%) | 447 (99.6%) | 2 (0.4%) | 0 (0.0%) |
| rs1043994 | 267 (76.9%) | 73 (21%) | 7 (2%) | 345 (76.8%) | 97 (21.6%) | 7 (1.6%) |
| rs114457076 | 349 (100%) | 0 (0.0%) | 0 (0.0%) | 448 (99.8%) | 1 (0.2%) | 0 (0.0%) |
| rs116239440 | 349 (100%) | 0 (0.0%) | 0 (0.0%) | 447 (99.6%) | 2 (0.4%) | 0 (0.0%) |
| rs61749020 | 326 (94.2%) | 20 (5.8%) | 0 (0.0%) | 416 (93.3%) | 29 (6.5%) | 1 (0.2%) |
| rs11670799 | 283 (99%) | 3 (1%) | 0 (0.0%) | 413 (96.5%) | 15 (3.5%) | 0 (0.0%) |
| rs114207045 | 346 (99.1%) | 3 (0.9%) | 0 (0.0%) | 445 (99.1%) | 4 (0.9%) | 0 (0.0%) |
| rs142762020 | 347 (100%) | 0 (0.0%) | 0 (0.0%) | 452 (100%) | 0 (0.0%) | 0 (0.0%) |
| rs146055867 | 348 (100%) | 0 (0.0%) | 0 (0.0%) | 447 (99.6%) | 2 (0.4%) | 0 (0.0%) |
| ss153922421 | 309 (100%) | 0 (0.0%) | 0 (0.0%) | 436 (100%) | 0 (0.0%) | 0 (0.0%) |
| rs79926127 | 307 (99.7%) | 1 (0.3%) | 0 (0.0%) | 429 (98.4%) | 7 (1.6%) | 0 (0.0%) |
| rs35793356 | 347 (100%) | 0 (0.0%) | 0 (0.0%) | 451 (99.8%) | 1 (0.2%) | 0 (0.0%) |
| rs140040122 | 346 (99.7%) | 1 (0.3%) | 0 (0.0%) | 451 (99.8%) | 1 (0.2%) | 0 (0.0%) |
| rs1043996 | 182 (52.8%) | 139 (40.3%) | 24 (7%) | 230 (51.6%) | 186 (41.7%) | 30 (6.7%) |
| rs1043997 | 262 (75.5%) | 78 (22.5%) | 7 (2%) | 336 (75%) | 105 (23.4%) | 7 (1.6%) |
| rs35769976 | 341 (97.7%) | 8 (2.3%) | 0 (0.0%) | 441 (98.2%) | 8 (1.8%) | 0 (0.0%) |
| rs146829488 | 347 (100%) | 0 (0.0%) | 0 (0.0%) | 452 (100%) | 0 (0.0%) | 0 (0.0%) |
| rs140642726 | 347 (100%) | 0 (0.0%) | 0 (0.0%) | 452 (100%) | 0 (0.0%) | 0 (0.0%) |
| rs112197217 | 334 (95.7%) | 14 (4%) | 1 (0.3%) | 431 (96%) | 18 (4%) | 0 (0.0%) |
| rs10408676 | 344 (98.6%) | 5 (1.4%) | 0 (0.0%) | 446 (99.3%) | 3 (0.7%) | 0 (0.0%) |
| rs1044006 | 284 (82.8%) | 57 (16.6%) | 2 (0.6%) | 363 (81.2%) | 79 (17.7%) | 5 (1.1%) |
| rs150037063 | 346 (99.4%) | 2 (0.6%) | 0 (0.0%) | 448 (99.8%) | 1 (0.2%) | 0 (0.0%) |
| rs78501403 | 294 (93.3%) | 21 (6.7%) | 0 (0.0%) | 402 (94.8%) | 22 (5.2%) | 0 (0.0%) |
| rs149222385 | 349 (100%) | 0 (0.0%) | 0 (0.0%) | 448 (99.8%) | 1 (0.2%) | 0 (0.0%) |
| rs143411026 | 349 (100%) | 0 (0.0%) | 0 (0.0%) | 448 (99.8%) | 1 (0.2%) | 0 (0.0%) |
| rs16980398 | 343 (98.3%) | 6 (1.7%) | 0 (0.0%) | 443 (98.9%) | 5 (1.1%) | 0 (0.0%) |
| rs115582213 | 342 (98%) | 7 (2%) | 0 (0.0%) | 438 (97.6%) | 11 (2.4%) | 0 (0.0%) |
| rs145859816 | 345 (100%) | 0 (0.0%) | 0 (0.0%) | 450 (100%) | 0 (0.0%) | 0 (0.0%) |
| rs114447350 | 260 (99.6%) | 1 (0.4%) | 0 (0.0%) | 406 (99.5%) | 2 (0.5%) | 0 (0.0%) |
| rs141231747 | 345 (100%) | 0 (0.0%) | 0 (0.0%) | 446 (100%) | 0 (0.0%) | 0 (0.0%) |
| rs1044008 | 322 (92.5%) | 23 (6.6%) | 3 (0.9%) | 412 (91.8%) | 33 (7.3%) | 4 (0.9%) |
| rs1044009 | 183 (62.2%) | 95 (32.3%) | 16 (5.4%) | 246 (59%) | 157 (37.6%) | 14 (3.4%) |
| rs61731975 | 346 (99.7%) | 1 (0.3%) | 0 (0.0%) | 446 (99.6%) | 2 (0.4%) | 0 (0.0%) |
| rs61731974 | 347 (100%) | 0 (0.0%) | 0 (0.0%) | 448 (100%) | 0 (0.0%) | 0 (0.0%) |
