# Supplementary material for: NOTCH3 Variants and Risk of Ischemic Stroke
Source: PLoS One. 2013 Sep 23;8(9):e75035. doi: 10.1371/journal.pone.0075035 (PMC3781028; doi:10.1371/journal.pone.0075035)
Supplement: Table S6 — Genotype frequencies in the combined Caucasian series. (DOCX) [file pone.0075035.s007.docx]

**Table S6: Genotype frequencies in the combined Caucasian series**

|  | Controls (N=1,004) | | | Stroke patients (N=721) | | |
| --- | --- | --- | --- | --- | --- | --- |
| SNP | Major/Major | Major/Minor | Minor/Minor | Major/Major | Major/Minor | Minor/Minor |
| rs3815188 | 708 (71.6%) | 270 (27.3%) | 11 (1.1%) | 503 (70%) | 201 (28%) | 15 (2.1%) |
| rs147373451 | 996 (99.3%) | 7 (0.7%) | 0 (0.0%) | 711 (99.6%) | 3 (0.4%) | 0 (0.0%) |
| rs1043994 | 766 (77.1%) | 201 (20.2%) | 27 (2.7%) | 554 (77.2%) | 152 (21.2%) | 12 (1.7%) |
| rs114457076 | 1001 (99.8%) | 2 (0.2%) | 0 (0.0%) | 713 (99.6%) | 3 (0.4%) | 0 (0.0%) |
| rs116239440 | 1002 (99.9%) | 1 (0.1%) | 0 (0.0%) | 712 (99.4%) | 4 (0.6%) | 0 (0.0%) |
| rs61749020 | 917 (92.9%) | 70 (7.1%) | 0 (0.0%) | 663 (94%) | 41 (5.8%) | 1 (0.1%) |
| rs11670799 | 882 (96.7%) | 30 (3.3%) | 0 (0.0%) | 678 (97.3%) | 19 (2.7%) | 0 (0.0%) |
| rs114207045 | 995 (99.2%) | 8 (0.8%) | 0 (0.0%) | 709 (99%) | 7 (1%) | 0 (0.0%) |
| rs142762020 | 995 (100%) | 0 (0.0%) | 0 (0.0%) | 720 (99.9%) | 1 (0.1%) | 0 (0.0%) |
| rs146055867 | 999 (99.7%) | 3 (0.3%) | 0 (0.0%) | 713 (99.6%) | 3 (0.4%) | 0 (0.0%) |
| ss153922421 | 943 (100%) | 0 (0.0%) | 0 (0.0%) | 704 (99.9%) | 1 (0.1%) | 0 (0.0%) |
| rs79926127 | 932 (98.9%) | 10 (1.1%) | 0 (0.0%) | 692 (98.2%) | 13 (1.8%) | 0 (0.0%) |
| rs35793356 | 993 (99.8%) | 2 (0.2%) | 0 (0.0%) | 718 (99.6%) | 3 (0.4%) | 0 (0.0%) |
| rs140040122 | 990 (99.6%) | 4 (0.4%) | 0 (0.0%) | 719 (99.7%) | 2 (0.3%) | 0 (0.0%) |
| rs1043996 | 491 (49.6%) | 412 (41.6%) | 87 (8.8%) | 352 (49.4%) | 295 (41.4%) | 65 (9.1%) |
| rs1043997 | 734 (73.6%) | 233 (23.4%) | 30 (3%) | 534 (74.8%) | 167 (23.4%) | 13 (1.8%) |
| rs35769976 | 968 (96.6%) | 30 (3%) | 4 (0.4%) | 697 (97.3%) | 19 (2.7%) | 0 (0.0%) |
| rs146829488 | 995 (100%) | 0 (0.0%) | 0 (0.0%) | 720 (99.9%) | 1 (0.1%) | 0 (0.0%) |
| rs140642726 | 994 (100%) | 0 (0.0%) | 0 (0.0%) | 720 (99.9%) | 1 (0.1%) | 0 (0.0%) |
| rs112197217 | 975 (97.2%) | 27 (2.7%) | 1 (0.1%) | 691 (96.5%) | 25 (3.5%) | 0 (0.0%) |
| rs10408676 | 977 (97.4%) | 24 (2.4%) | 2 (0.2%) | 707 (98.7%) | 9 (1.3%) | 0 (0.0%) |
| rs1044006 | 803 (81.8%) | 165 (16.8%) | 14 (1.4%) | 585 (81.8%) | 124 (17.3%) | 6 (0.8%) |
| rs150037063 | 998 (99.6%) | 4 (0.4%) | 0 (0.0%) | 714 (99.7%) | 2 (0.3%) | 0 (0.0%) |
| rs78501403 | 860 (91.4%) | 81 (8.6%) | 0 (0.0%) | 662 (95.9%) | 28 (4.1%) | 0 (0.0%) |
| rs149222385 | 1002 (100%) | 0 (0.0%) | 0 (0.0%) | 714 (99.7%) | 2 (0.3%) | 0 (0.0%) |
| rs143411026 | 1003 (100%) | 0 (0.0%) | 0 (0.0%) | 714 (99.7%) | 2 (0.3%) | 0 (0.0%) |
| rs16980398 | 973 (97%) | 25 (2.5%) | 5 (0.5%) | 701 (98%) | 14 (2%) | 0 (0.0%) |
| rs115582213 | 977 (97.4%) | 26 (2.6%) | 0 (0.0%) | 700 (97.8%) | 16 (2.2%) | 0 (0.0%) |
| rs145859816 | 993 (100%) | 0 (0.0%) | 0 (0.0%) | 718 (99.9%) | 1 (0.1%) | 0 (0.0%) |
| rs114447350 | 853 (99.5%) | 4 (0.5%) | 0 (0.0%) | 673 (99.4%) | 4 (0.6%) | 0 (0.0%) |
| rs141231747 | 989 (100%) | 0 (0.0%) | 0 (0.0%) | 711 (99.9%) | 1 (0.1%) | 0 (0.0%) |
| rs1044008 | 921 (91.9%) | 77 (7.7%) | 4 (0.4%) | 651 (90.9%) | 60 (8.4%) | 5 (0.7%) |
| rs1044009 | 555 (61%) | 304 (33.4%) | 51 (5.6%) | 396 (57.8%) | 253 (36.9%) | 36 (5.3%) |
| rs61731975 | 996 (99.5%) | 5 (0.5%) | 0 (0.0%) | 710 (99.3%) | 5 (0.7%) | 0 (0.0%) |
| rs61731974 | 998 (99.7%) | 3 (0.3%) | 0 (0.0%) | 714 (99.9%) | 1 (0.1%) | 0 (0.0%) |
