# Supplementary material for: NOTCH3 Variants and Risk of Ischemic Stroke
Source: PLoS One. 2013 Sep 23;8(9):e75035. doi: 10.1371/journal.pone.0075035 (PMC3781028; doi:10.1371/journal.pone.0075035)
Supplement: Table S7 — Genotype frequencies in the ISGS African American series. (DOCX) [file pone.0075035.s008.docx]

**Table S7: Genotype frequencies in the ISGS African American series**

|  | Controls (N=131) | | | Stroke patients (N=167) | | |
| --- | --- | --- | --- | --- | --- | --- |
| SNP | Major/Major | Major/Minor | Minor/Minor | Major/Major | Major/Minor | Minor/Minor |
| rs3815188 | 72 (56.2%) | 43 (33.6%) | 13 (10.2%) | 86 (52.1%) | 68 (41.2%) | 11 (6.7%) |
| rs147373451 | 131 (100%) | 0 (0.0%) | 0 (0.0%) | 164 (100%) | 0 (0.0%) | 0 (0.0%) |
| rs1043994 | 106 (80.9%) | 22 (16.8%) | 3 (2.3%) | 139 (83.2%) | 27 (16.2%) | 1 (0.6%) |
| rs114457076 | 131 (100%) | 0 (0.0%) | 0 (0.0%) | 164 (100%) | 0 (0.0%) | 0 (0.0%) |
| rs116239440 | 131 (100%) | 0 (0.0%) | 0 (0.0%) | 164 (100%) | 0 (0.0%) | 0 (0.0%) |
| rs61749020 | 126 (96.9%) | 4 (3.1%) | 0 (0.0%) | 157 (94.6%) | 9 (5.4%) | 0 (0.0%) |
| rs11670799 | 97 (99%) | 1 (1%) | 0 (0.0%) | 160 (100%) | 0 (0.0%) | 0 (0.0%) |
| rs114207045 | 128 (97.7%) | 3 (2.3%) | 0 (0.0%) | 162 (98.8%) | 2 (1.2%) | 0 (0.0%) |
| rs142762020 | 131 (100%) | 0 (0.0%) | 0 (0.0%) | 167 (100%) | 0 (0.0%) | 0 (0.0%) |
| rs146055867 | 131 (100%) | 0 (0.0%) | 0 (0.0%) | 164 (100%) | 0 (0.0%) | 0 (0.0%) |
| ss153922421 | 107 (100%) | 0 (0.0%) | 0 (0.0%) | 164 (100%) | 0 (0.0%) | 0 (0.0%) |
| rs79926127 | 104 (97.2%) | 3 (2.8%) | 0 (0.0%) | 164 (100%) | 0 (0.0%) | 0 (0.0%) |
| rs35793356 | 126 (96.2%) | 5 (3.8%) | 0 (0.0%) | 156 (93.4%) | 11 (6.6%) | 0 (0.0%) |
| rs140040122 | 131 (100%) | 0 (0.0%) | 0 (0.0%) | 167 (100%) | 0 (0.0%) | 0 (0.0%) |
| rs1043996 | 73 (57%) | 42 (32.8%) | 13 (10.2%) | 88 (53.7%) | 66 (40.2%) | 10 (6.1%) |
| rs1043997 | 57 (44.2%) | 53 (41.1%) | 19 (14.7%) | 63 (38.7%) | 76 (46.6%) | 24 (14.7%) |
| rs35769976 | 73 (55.7%) | 47 (35.9%) | 11 (8.4%) | 80 (48.8%) | 69 (42.1%) | 15 (9.1%) |
| rs146829488 | 131 (100%) | 0 (0.0%) | 0 (0.0%) | 167 (100%) | 0 (0.0%) | 0 (0.0%) |
| rs140642726 | 131 (100%) | 0 (0.0%) | 0 (0.0%) | 167 (100%) | 0 (0.0%) | 0 (0.0%) |
| rs112197217 | 131 (100%) | 0 (0.0%) | 0 (0.0%) | 163 (99.4%) | 1 (0.6%) | 0 (0.0%) |
| rs10408676 | 80 (61.1%) | 40 (30.5%) | 11 (8.4%) | 94 (57.3%) | 62 (37.8%) | 8 (4.9%) |
| rs1044006 | 121 (95.3%) | 6 (4.7%) | 0 (0.0%) | 158 (95.8%) | 7 (4.2%) | 0 (0.0%) |
| rs150037063 | 131 (100%) | 0 (0.0%) | 0 (0.0%) | 164 (100%) | 0 (0.0%) | 0 (0.0%) |
| rs78501403 | 106 (93%) | 8 (7%) | 0 (0.0%) | 146 (90.7%) | 14 (8.7%) | 1 (0.6%) |
| rs149222385 | 131 (100%) | 0 (0.0%) | 0 (0.0%) | 164 (100%) | 0 (0.0%) | 0 (0.0%) |
| rs143411026 | 131 (100%) | 0 (0.0%) | 0 (0.0%) | 164 (100%) | 0 (0.0%) | 0 (0.0%) |
| rs16980398 | 59 (45%) | 50 (38.2%) | 22 (16.8%) | 61 (37.2%) | 83 (50.6%) | 20 (12.2%) |
| rs115582213 | 131 (100%) | 0 (0.0%) | 0 (0.0%) | 164 (100%) | 0 (0.0%) | 0 (0.0%) |
| rs145859816 | 131 (100%) | 0 (0.0%) | 0 (0.0%) | 167 (100%) | 0 (0.0%) | 0 (0.0%) |
| rs114447350 | 83 (86.5%) | 13 (13.5%) | 0 (0.0%) | 124 (81.6%) | 28 (18.4%) | 0 (0.0%) |
| rs141231747 | 131 (100%) | 0 (0.0%) | 0 (0.0%) | 167 (100%) | 0 (0.0%) | 0 (0.0%) |
| rs1044008 | 129 (98.5%) | 2 (1.5%) | 0 (0.0%) | 163 (99.4%) | 1 (0.6%) | 0 (0.0%) |
| rs1044009 | 28 (29.5%) | 48 (50.5%) | 19 (20%) | 47 (30.5%) | 72 (46.8%) | 35 (22.7%) |
| rs61731975 | 108 (83.1%) | 21 (16.2%) | 1 (0.8%) | 137 (83.5%) | 26 (15.9%) | 1 (0.6%) |
| rs61731974 | 125 (96.2%) | 4 (3.1%) | 1 (0.8%) | 147 (89.6%) | 17 (10.4%) | 0 (0.0%) |
